# Data from uniaxial compressive testing of laboratory-made granular ice

**DOI:** 10.1016/j.dib.2022.108236

**Published:** 2022-05-04

**Authors:** Angelo Mario Böhm, Hauke Herrnring, Franz von Bock und Polach

**Affiliations:** Hamburg University of Technology, Institute for Ship Structural Design and Analysis, 21073, Hamburg, Germany

**Keywords:** Uniaxial compression, Ice, Ductile, Brittle, Axial splitting, Displacement controlled test

## Abstract

Uniaxial compressive tests of laboratory-made granular fresh-water ice were conducted in a cold room in the ductile and brittle strain rate range at -10.83°C ±0.74°C. Ice specimens with a length to diameter ratio of 2.5 showing brittle behavior failed by axial splitting. With the *Instron Labtronic 8800*, the operator controlled the tests at a frequency of 4,000 Hz. The data acquisition rate was 25,000 Hz, and for faster experiments, 100,000 Hz. The operator controlled on a random basis the hydraulic cylinder by either the cylinder displacement or the specimen displacement. Increasing as well as constant and decreasing compression strength trends with increasing strain rates could be shown in the past. The data presented here show a lower compressive strength at strain rates higher than 4*10^−3^ s^−1^. The data consist of the time history of the specimen and cylinder displacement measurement (in mm), and the force measurement (in kN). The data is available as a separate *.xlsx* file for each test performed. In total, 123 tests were performed. If the test was performed with a 10 mm gap, the label ends with a ‘*g’*. The abbreviations are separated with an underscore.

The data provided here can be used to validate ice-material models or for ice-testing databases for machine learning purposes.

## Specifications Table

**Table:** 

Subject	Materials Science
Specific subject area	Uniaxial compression test of ice
Type of data	Table, *xlsx* file
How the data were acquired	Force measurement through a *Schenck PM160Rn* load cell, cylinder displacement measurement through the Schenck *Hydropuls-Longitudinal PL 160* cylinder, which uses a build-in inductive displacement sensor, and specimen displacement measurement through the potentiometric displacement sensor *burster 8718-500* controlled with the *Instron Labtronic 8800*. The measurements are carried out with the *Gantner Q.raxx-station 101 T* measuring amplifier. The displacement, the force and the temperature are measured with the *Gantner Q.raxx A101* module.
Data format	Raw and processed
Description of data collection	The data acquisition rate was 25,000 Hz for test series 1 – 3 and 100,000 Hz for tests series 4. The sample rate was decreased in the post process for test with a test velocity between 0.01 mm/s to 0.1 mm/s by a factor 100 to decrease the file size.
Data source location	• Institution: Hamburg University of Technology • City/Town/Region: Hamburg • Country: Germany • Latitude and longitude: 53°27′46.8″N 9°58′11.8″E
Data accessibility	Repository name: Mendeley Data identification number: 10.17632/m4s8vkd8c5.3 Direct link to the dataset: https://data.mendeley.com/datasets/m4s8vkd8c5/3 Mendeley data: Version 3

## Value of the Data

• The dataset is relevant as it is a complete presentation of uniaxial compression tests of granular ice at a high degree of detail.
• Experimenters can benefit from the data, as it shows influences of the testing properties on the uniaxial compression tests of ice over a wide range of testing velocities/strain rates, which was not provided before at this degree of detail.
• Data scientists with a material modeling background can benefit from the detailed data, as it shows influences of the strain rate on the uniaxial compressive strength of ice.
• The presented data can be used to validate numerical ice material models or for data-driven analyses and modeling of uniaxial compression tests of ice.

## 1 Data Description

The data being shared describe the uniaxial compression properties of laboratory-made granular ice. Table 1, Table 2, Table 3, Table 4 show the testing settings: Set velocity for the compression test, if the operator controlled the test with the cylinder displacement (CDC) or with the specimen displacement (SDC), the PID controller settings, the data acquisition rate and the repository file name where the time history of the force and displacement measurement is stored. In the post-process of the data acquisition, the data was cut, and for tests with set velocities between 0.01 mm/s to 0.1 mm/s the sample rate was decreased by a factor of 100 to decrease the file size. The start point of the cutting process was a measured force of 1 kN, and the endpoint was either the maximum measured force for tests showing brittle behavior or a measured displacement around 10 mm for tests showing ductile behavior. The zero point of the time and displacement measurement was shifted to the start point, also defined as the point of contact.

**Table 1:** Series 1 - Uniaxial compression settings and data of granular ice.

				PID settings				

Date	No.	Set velocity [mm/s]	Controlling	P [dB]	I [1/s]	D [ms]	Sampling rate [kHz]	Repository file name
22.09.2021	1	1	SDC	15	1	0	25	2021_09_22_1_v1_SDC.xlsx
22.09.2021	2	1	SDC	15	1	0	25	2021_09_22_2_v1_SDC.xlsx
22.09.2021	3	0.01	SDC	15	1	0	25	2021_09_22_3_v001_SDC.xlsx
22.09.2021	4	0.06	SDC	15	1	0	25	2021_09_22_4_v006_SDC.xlsx
22.09.2021	5	0.05	SDC	15	1	0	25	2021_09_22_5_v005_SDC.xlsx
22.09.2021	6	1	SDC	15	1	0	25	2021_09_22_6_v1_SDC.xlsx
23.09.2021	7	0.01	SDC	15	1	0	25	2021_09_23_7_v001_SDC.xlsx
23.09.2021	8	0.01	CDC	19.1	0.1	0.3	25	2021_09_23_8_v001_CDC.xlsx
23.09.2021	9	1	SDC	15	1	0	25	2021_09_23_9_v1_SDC.xlsx
23.09.2021	10	1	SDC	15	1	0	25	2021_09_23_10_v1_SDC.xlsx
23.09.2021	11	0.05	CDC	19.1	0.1	0.3	25	2021_09_23_11_v005_CDC.xlsx
23.09.2021	12	0.01	SDC	15	1	0	25	2021_09_23_12_v001_SDC.xlsx
24.09.2021	13	0.01	CDC	19.1	0.1	0.3	25	2021_09_24_13_v001_CDC.xlsx
24.09.2021	15	0.06	CDC	19.1	0.1	0.3	25	2021_09_24_15_v006_CDC.xlsx
24.09.2021	16	1	CDC	19.1	0.1	0.3	25	2021_09_24_16_v1_CDC.xlsx
24.09.2021	17	0.06	CDC	19.1	0.1	0.3	25	2021_09_24_17_v006_CDC.xlsx
24.09.2021	18	0.01	SDC	15	1	0	25	2021_09_24_18_v001_SDC.xlsx
24.09.2021	19	0.05	CDC	19.1	0.1	0.3	25	2021_09_24_19_v005_CDC.xlsx
24.09.2021	20	0.01	CDC	19.1	0.1	0.3	25	2021_09_24_20_v001_CDC.xlsx
24.09.2021	21	0.01	CDC	19.1	0.1	0.3	25	2021_09_24_21_v001_CDC.xlsx
24.09.2021	22	0.06	CDC	19.1	0.1	0.3	25	2021_09_24_22_v006_CDC.xlsx
27.09.2021	23	0.01	CDC	19.1	0.1	0.3	25	2021_09_27_23_v001_CDC.xlsx
27.09.2021	24	0.01	SDC	15	1	0	25	2021_09_27_24_v001_SDC.xlsx
27.09.2021	25	0.05	SDC	15	1	0	25	2021_09_27_25_v005_SDC.xlsx
27.09.2021	26	0.05	SDC	15	1	0	25	2021_09_27_26_v005_SDC.xlsx
27.09.2021	27	0.06	SDC	15	1	0	25	2021_09_27_27_v006_SDC.xlsx
27.09.2021	28	1	SDC	15	1	0	25	2021_09_27_28_v1_SDC.xlsx
27.09.2021	29	1	SDC	15	1	0	25	2021_09_27_29_v1_SDC.xlsx
27.09.2021	30	1	SDC	15	1	0	25	2021_09_27_30_v1_SDC.xlsx
27.09.2021	31	0.06	CDC	19.1	0.1	0.3	25	2021_09_27_31_v006_CDC.xlsx
27.09.2021	32	1	SDC	15	1	0	25	2021_09_27_32_v1_SDC.xlsx
27.09.2021	33	1	SDC	15	1	0	25	2021_09_27_33_v1_SDC.xlsx

**Table 2:** Series 2 - Uniaxial compression settings and data of granular ice.

				PID settings				

Date	No.	Set velocity [mm/s]	Controlling	P [dB]	I [1/s]	D [ms]	Sampling rate [kHz]	Repository file name
14.10.2021	1	100	SDC	21.5	0.1	1.4	25	2021_10_14_1_v100_SDC.xlsx
14.10.2021	2	1	SDC	21.5	0.1	1.4	25	2021_10_14_2_v1_SDC.xlsx
14.10.2021	3	100	SDC	21.5	0.1	1.4	25	2021_10_14_3_v100_SDC.xlsx
14.10.2021	4	10	SDC	21.5	0.1	1.4	25	2021_10_14_4_v10_SDC.xlsx
14.10.2021	5	1	SDC	21.5	0.1	1.4	25	2021_10_14_5_v1_SDC.xlsx
14.10.2021	6	100	SDC	21.5	0.1	1.4	25	2021_10_14_6_v100_SDC.xlsx
14.10.2021	7	1	SDC	21.5	0.1	1.4	25	2021_10_14_7_v1_SDC.xlsx
14.10.2021	8	1	SDC	21.5	0.1	1.4	25	2021_10_14_8_v1_SDC.xlsx
15.10.2021	9	100	SDC	22.7	0.1	1.5	25	2021_10_15_9_v100_SDC.xlsx
15.10.2021	10	1	SDC	22.7	0.1	1.5	25	2021_10_15_10_v1_SDC.xlsx
15.10.2021	11	100	SDC	22.7	0.1	1.5	25	2021_10_15_11_v100_SDC.xlsx
15.10.2021	12	1	SDC	22.7	0.1	1.5	25	2021_10_15_12_v1_SDC.xlsx
15.10.2021	13	1	SDC	22.7	0.1	1.5	25	2021_10_15_13_v1_SDC.xlsx
15.10.2021	14	10	SDC	22.7	0.1	1.5	25	2021_10_15_14_v10_SDC.xlsx
15.10.2021	15	100	SDC	22.7	0.1	1.5	25	2021_10_15_15_v100_SDC.xlsx
15.10.2021	16	100	SDC	22.7	0.1	1.5	25	2021_10_15_16_v100_SDC.xlsx
15.10.2021	17	10	SDC	22.7	0.1	1.5	25	2021_10_15_17_v10_SDC.xlsx
15.10.2021	18	1	SDC	22.7	0.1	1.5	25	2021_10_15_18_v1_SDC.xlsx
15.10.2021	19	10	SDC	22.7	0.1	1.5	25	2021_10_15_19_v10_SDC.xlsx
15.10.2021	20	1	SDC	22.7	0.1	1.5	25	2021_10_15_20_v1_SDC.xlsx
15.10.2021	21	10	SDC	22.7	0.1	1.5	25	2021_10_15_21_v10_SDC.xlsx
15.10.2021	22	100	SDC	22.7	0.1	1.5	25	2021_10_15_22_v100_SDC.xlsx
15.10.2021	23	10	SDC	22.7	0.1	1.5	25	2021_10_15_23_v10_SDC.xlsx
15.10.2021	24	10	SDC	22.7	0.1	1.5	25	2021_10_15_24_v10_SDC.xlsx
15.10.2021	25	10	SDC	22.7	0.1	1.5	25	2021_10_15_25_v10_SDC.xlsx
15.10.2021	26	100	SDC	22.7	0.1	1.5	25	2021_10_15_26_v100_SDC.xlsx
15.10.2021	27	100	SDC	22.7	0.1	1.5	25	2021_10_15_27_v100_SDC.xlsx

**Table 3:** Series 3 - Uniaxial compression settings and data of granular ice.

				PID settings				

Date	No.	Set velocity [mm/s]	Controlling	P [dB]	I [1/s]	D [ms]	Sampling rate [kHz]	Repository file name
22.11.2021	1	10	CDC	22.7	0.1	1.5	25	2021_11_22_1_v10_CDC.xlsx
22.11.2021	2	100	SDC	22.7	0.1	1.5	25	2021_11_22_2_v100_SDC.xlsx
22.11.2021	3	100	CDC	22.7	0.1	1.5	25	2021_11_22_3_v100_CDC.xlsx
22.11.2021	4	1	CDC	22.7	0.1	1.5	25	2021_11_22_4_v1_CDC.xlsx
22.11.2021	5	100	SDC	22.7	0.1	1.5	25	2021_11_22_5_v100_SDC.xlsx
22.11.2021	6	10	SDC	22.7	0.1	1.5	25	2021_11_22_6_v10_SDC.xlsx
22.11.2021	7	0.1	SDC	22.7	0.1	1.5	25	2021_11_22_7_v01_SDC.xlsx
22.11.2021	8	100	SDC	22.7	0.1	1.5	25	2021_11_22_8_v100_SDC.xlsx
22.11.2021	9	10	CDC	22.7	0.1	1.5	25	2021_11_22_9_v10_CDC.xlsx
22.11.2021	10	100	CDC	22.7	0.1	1.5	25	2021_11_22_10_v100_CDC.xlsx
22.11.2021	11	10	SDC	22.7	0.1	1.5	25	2021_11_22_11_v10_SDC.xlsx
22.11.2021	12	0.1	CDC	22.7	0.1	1.5	25	2021_11_22_12_v01_CDC.xlsx
22.11.2021	13	10	SDC	22.7	0.1	1.5	25	2021_11_22_13_v10_SDC.xlsx
22.11.2021	14	100	CDC	22.7	0.1	1.5	25	2021_11_22_14_v100_CDC.xlsx
22.11.2021	15	100	SDC	22.7	0.1	1.5	25	2021_11_22_15_v100_SDC.xlsx
22.11.2021	16	10	CDC	22.7	0.1	1.5	25	2021_11_22_16_v10_CDC.xlsx
22.11.2021	17	100	SDC	22.7	0.1	1.5	25	2021_11_22_17_v100_SDC.xlsx
22.11.2021	18	100	SDC	22.7	0.1	1.5	25	2021_11_22_18_v100_SDC.xlsx
22.11.2021	19	1	SDC	22.7	0.1	1.5	25	2021_11_22_19_v1_SDC.xlsx
22.11.2021	20	1	SDC	22.7	0.1	1.5	25	2021_11_22_20_v1_SDC.xlsx
22.11.2021	21	10	SDC	22.7	0.1	1.5	25	2021_11_22_21_v10_SDC.xlsx
22.11.2021	22	100	CDC	22.7	0.1	1.5	25	2021_11_22_22_v100_CDC.xlsx
22.11.2021	23	1	SDC	22.7	0.1	1.5	25	2021_11_22_23_v1_SDC.xlsx
22.11.2021	24	10	CDC	22.7	0.1	1.5	25	2021_11_22_24_v10_CDC.xlsx
22.11.2021	25	1	CDC	22.7	0.1	1.5	25	2021_11_22_25_v1_CDC.xlsx
22.11.2021	26	0.1	SDC	22.7	0.1	1.5	25	2021_11_22_26_v01_SDC.xlsx
22.11.2021	27	10	CDC	22.7	0.1	1.5	25	2021_11_22_27_v10_CDC.xlsx
22.11.2021	28	100	CDC	22.7	0.1	1.5	25	2021_11_22_28_v100_CDC.xlsx
22.11.2021	29	10	SDC	22.7	0.1	1.5	25	2021_11_22_29_v10_SDC.xlsx
22.11.2021	30	10	SDC	22.7	0.1	1.5	25	2021_11_22_30_v10_SDC.xlsx
22.11.2021	31	0.1	CDC	22.7	0.1	1.5	25	2021_11_22_31_v01_CDC.xlsx

**Table 4:** Series 4 - Uniaxial compression settings and data of granular ice.

				PID settings				

Date	No.	Set velocity [mm/s]	Controlling	P [dB]	I [1/s]	D [ms]	Sampling rate [kHz]	Repository file name
30.11.2021	1	100	CDC	22.7	0.1	1.5	100	2021_11_30_1_v100_CDC_g.xlsx
30.11.2021	2	10	CDC	22.7	0.1	1.5	100	2021_11_30_2_v10_CDC.xlsx
30.11.2021	3	100	SDC	22.7	0.1	1.5	100	2021_11_30_3_v100_SDC_g.xlsx
30.11.2021	4	100	SDC	22.7	0.1	1.5	100	2021_11_30_4_v100_SDC.xlsx
30.11.2021	5	10	SDC	22.7	0.1	1.5	100	2021_11_30_5_v10_SDC.xlsx
30.11.2021	6	10	SDC	22.7	0.1	1.5	100	2021_11_30_6_v10_SDC_g.xlsx
30.11.2021	7	1	SDC	22.7	0.1	1.5	100	2021_11_30_7_v1_SDC.xlsx
30.11.2021	8	1	CDC	22.7	0.1	1.5	100	2021_11_30_8_v1_CDC.xlsx
30.11.2021	9	100	SDC	22.7	0.1	1.5	100	2021_11_30_9_v100_SDC.xlsx
30.11.2021	10	1000	CDC	22.7	0.1	1.5	100	2021_11_30_10_v1000_CDC_g.xlsx
30.11.2021	11	10	CDC	22.7	0.1	1.5	100	2021_11_30_11_v10_CDC.xlsx
30.11.2021	12	10	SDC	22.7	0.1	1.5	100	2021_11_30_12_v10_SDC.xlsx
30.11.2021	13	100	SDC	22.7	0.1	1.5	100	2021_11_30_13_v100_SDC_g.xlsx
30.11.2021	14	10	CDC	22.7	0.1	1.5	100	2021_11_30_14_v10_CDC.xlsx
30.11.2021	15	500	SDC	22.7	0.1	1.5	100	2021_11_30_15_v500_SDC_g.xlsx
30.11.2021	16	500	SDC	22.7	0.1	1.5	100	2021_11_30_16_v500_SDC.xlsx
30.11.2021	17	500	SDC	22.7	0.1	1.5	100	2021_11_30_17_v500_SDC.xlsx
30.11.2021	18	500	SDC	22.7	0.1	1.5	100	2021_11_30_18_v500_SDC_g.xlsx
30.11.2021	19	100	CDC	22.7	0.1	1.5	100	2021_11_30_19_v100_CDC_g.xlsx
30.11.2021	20	500	CDC	22.7	0.1	1.5	100	2021_11_30_20_v500_CDC_g.xlsx
30.11.2021	21	500	SDC	22.7	0.1	1.5	100	2021_11_30_21_v500_SDC_g.xlsx
30.11.2021	22	100	SDC	22.7	0.1	1.5	100	2021_11_30_22_v100_SDC_g.xlsx
30.11.2021	23	100	CDC	22.7	0.1	1.5	100	2021_11_30_23_v100_CDC_g.xlsx
30.11.2021	24	100	CDC	22.7	0.1	1.5	100	2021_11_30_24_v100_CDC_g.xlsx
30.11.2021	25	500	CDC	22.7	0.1	1.5	100	2021_11_30_25_v500_CDC.xlsx
30.11.2021	26	100	CDC	22.7	0.1	1.5	100	2021_11_30_26_v100_CDC.xlsx
30.11.2021	27	1	CDC	22.7	0.1	1.5	100	2021_11_30_27_v1_CDC_g.xlsx
30.11.2021	28	500	CDC	22.7	0.1	1.5	100	2021_11_30_28_v500_CDC.xlsx
30.11.2021	29	500	CDC	22.7	0.1	1.5	100	2021_11_30_29_v500_CDC_g.xlsx
30.11.2021	30	10	SDC	22.7	0.1	1.5	100	2021_11_30_30_v10_SDC.xlsx
30.11.2021	31	1	SDC	22.7	0.1	1.5	100	2021_11_30_31_v1_SDC_g.xlsx
30.11.2021	32	10	CDC	22.7	0.1	1.5	100	2021_11_30_32_v10_CDC_g.xlsx
30.11.2021	33	0.5	CDC	22.7	0.1	1.5	100	2021_11_30_33_v05_CDC.xlsx

**Fig. 1:**
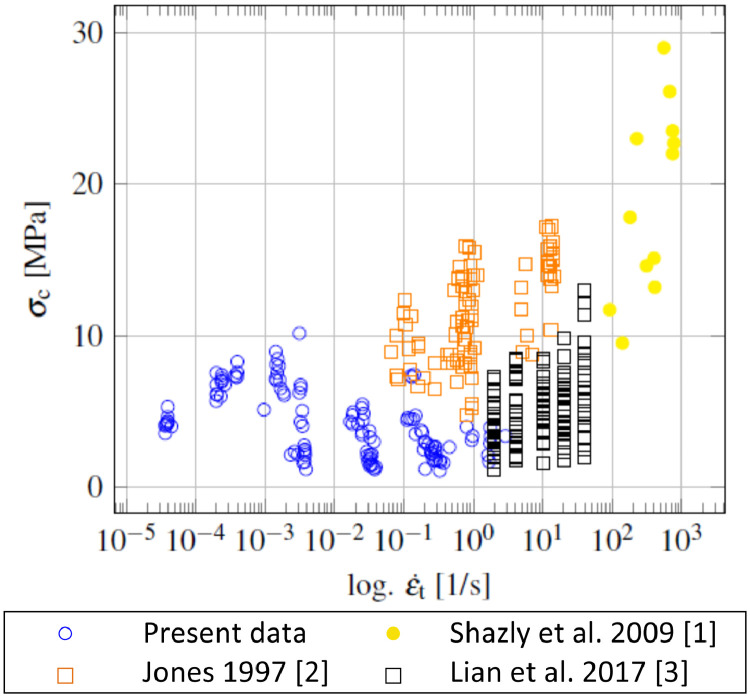
Uniaxial compressive strength of granular ice over strain rate; Present data and others.

Fig. 1 summarizes the uniaxial compressive strength of granular ice of the present data, besides some other studies.

Ice specimens with a length to diameter ratio of 2.5 showing brittle behavior failed by axial splitting. The data presented here show a lower compressive strength from strain rates higher than 4*10^−3^ s^−1^, compared to the other studies. The strength values in the brittle range are far below the strength values of Shazly et al. [1] and Jones [2]. The difference to Jones may be caused due to the different L/D ratio (2.08) and the columnar grain type, whereby Shazly has investigated single- and polycrystalline ice at much higher strain rates. Lian et al. [3] tested cubic, columnar ice specimens with a L/D ratio of 1 and presented the results over the apparent strain rate. Comparing different experimental ice compression studies is challenging as testing properties, ice specimen geometries, and grain types differ, and the indication of the fracture mode is sometimes lacking.

## 2 Experimental Design, Materials and Methods

The experimental design included the PID controller settings and the displacement control type and consisted of determining the compressive strength at high rates. The rate limitation was the Schenck Hydropuls-longitudinal PL 160 cylinder with its force capability of nominal 160 kN and a maximal velocity of 1 m/s. The hydraulic cylinder can be controlled either by the piston displacement (build-in inductive displacement sensor, CDC) or by the specimen displacement (SDC). The specimen displacement controlled tests neglect the effect of the test rig stiffness. All tests are performed at TUHH in the mechanical laboratory of the Institute for Ship Structural Design and Analysis.

### 2.1 Specimen production

Cylindrical ice specimens with a diameter of 99.4 mm and a total length of 250 mm were produced in an approximately -10°C cold refrigerated container.

PVC-U tubes, commercially available crushed ice, distilled water, steel plates, and insulating material were needed to make granular ice specimens. The distilled water had a mean acidity of 8.6 pH ±1 pH and a mean electrical conductivity of 7.5 μS/cm ± 5.8 μS/cm measured at a random basis at temperatures of 18.8°C ± 3.6°C.

**Fig. 2:**
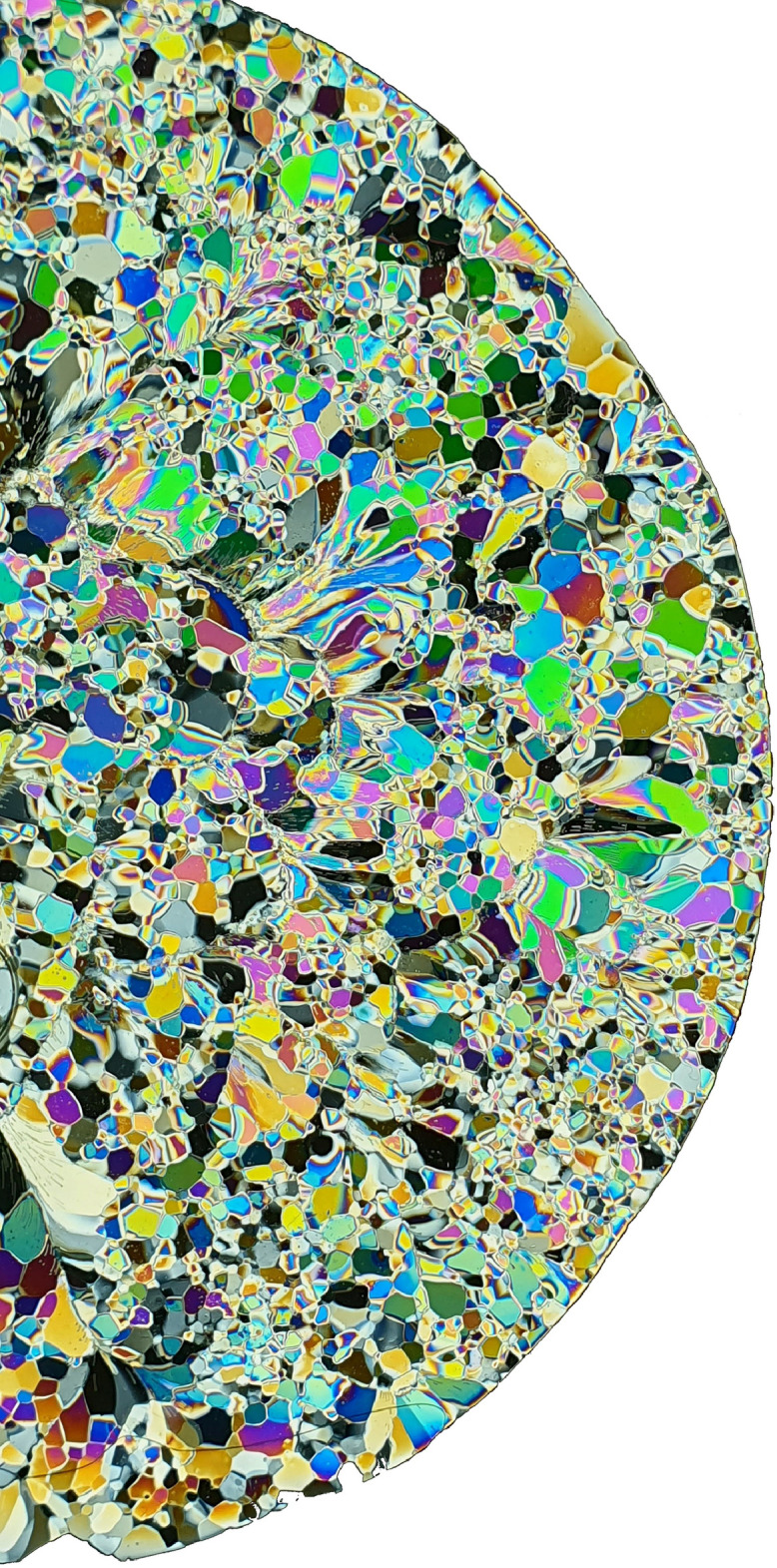
Microstructure of a specimen.

The tubes were cut into the desired length, including a minimum addition of 30 mm regarding the cutting process after the specimen removal, and glued to the steel plate on one side. The steel plate formed the base and ensured good heat conduction. In the refrigerated container, two-thirds of crushed ice and one-third of distilled water was added to the tube, and the top of the tube was covered with insulation material. After freezing for at least two days, the ice specimen was removed from the tube by heating it with a hot air gun up (about 2,000 W) for a short time. After removal, the ice specimens were stored in the refrigerated container and were cut at both ends into the desired length with a band saw located in the refrigerated container. This process ensured consistency in the grain structure and shape of the specimens. Fig. 2 shows a thin section of an exemplary specimen (grain size approximately 1-10 mm determined by manual counting).

Herrnring et al. [4,5] already used the ice production process successfully, based on Gudimetla [6], but in contrast, commercial crushed ice instead of self-made crushed ice was used.

### 2.2 Setup and methods

**Fig. 3:**
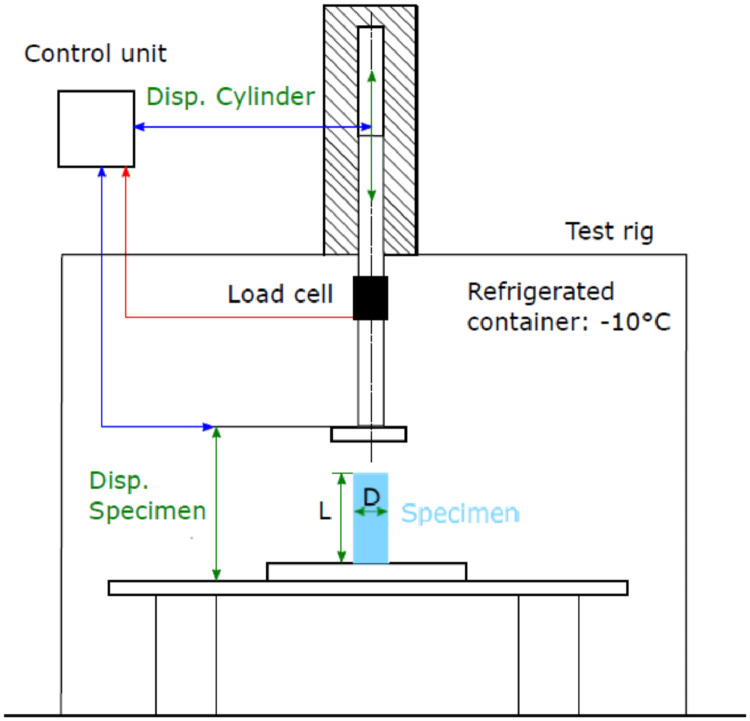
Test setup.

The *Schenk Hydropuls-Longitudinal PL 160 cylinder*, the position of the load cell, and the position of the potentiometric displacement sensor *burster 8718-500* for the specimen displacement measurement are shown in Fig. 3.

With the *Instron Labtronic 8800*, the operator controlled the tests at a frequency of 4,000 Hz. The measurements are carried out with the *Gantner Q.raxx-station 101 T* measuring amplifier. The displacement, the force and the temperature are measured with the *Q.raxx A101* module. The data acquisition rate was 25,000 Hz for test series 1 – 3 and 100,000 Hz for tests series 4, see also Table 1 to Table 4. Two operators were needed to perform the tests. One operator controlled the hydraulic cylinder, and the other operator started the measurement and prepared the measurement environment. The measurement environment had to be cleaned before each test, and the operator had to place the specimen centrally.

## CRediT Author Statement

**Angelo Mario Böhm:** Conceptualization, Methodology, Investigation, Data Curation, Writing – original draft, Writing – review & editing, Visualization; **Hauke Herrnring:** Software, Writing – review & editing; **Franz von Bock und Polach:** Supervision, Writing – review & editing.

## Declaration of competing interest

The authors declare that they have no known competing financial interests or personal relationships that could have appeared to influence the work reported in this paper.

## Data Availability

Data from uniaxial compressive testing of laboratory-made granular ice (Original data) (Mendeley Data).
